# Fast Iterative Shrinkage-Thresholding Algorithm with Continuation for Brain Injury Monitoring Imaging Based on Electrical Impedance Tomography

**DOI:** 10.3390/s22249934

**Published:** 2022-12-16

**Authors:** Xuechao Liu, Tao Zhang, Jian’an Ye, Xiang Tian, Weirui Zhang, Bin Yang, Meng Dai, Canhua Xu, Feng Fu

**Affiliations:** 1 Department of Biomedical Engineering, The Fourth Military Medical University, Xi’an 710032, China; 2 Shaanxi Key Laboratory for Bioelectromagnetic Detection and Intelligent Perception, Xi’an 710032, China; 3 Drug and Instrument Supervision and Inspection Station, Xining Joint Logistics Support Center, Lanzhou 730050, China

**Keywords:** electrical impedance tomography, reconstruction algorithm, brain injury monitoring imaging, fast iterative shrinkage-thresholding algorithm, least absolute shrinkage and selection operator model

## Abstract

Electrical impedance tomography (EIT) is low-cost and noninvasive and has the potential for real-time imaging and bedside monitoring of brain injury. However, brain injury monitoring by EIT imaging suffers from image noise (IN) and resolution problems, causing blurred reconstructions. To address these problems, a least absolute shrinkage and selection operator model is built, and a fast iterative shrinkage-thresholding algorithm with continuation (FISTA-C) is proposed. Results of numerical simulations and head phantom experiments indicate that FISTA-C reduces IN by 63.2%, 47.2%, and 29.9% and 54.4%, 44.7%, and 22.7%, respectively, when compared with the damped least-squares algorithm, the split Bergman, and the FISTA algorithms. When the signal-to-noise ratio of the measurements is 80–50 dB, FISTA-C can reduce IN by 83.3%, 72.3%, and 68.7% on average when compared with the three algorithms, respectively. Both simulation and phantom experiments suggest that FISTA-C produces the best image resolution and can identify the two closest targets. Moreover, FISTA-C is more practical for clinical application because it does not require excessive parameter adjustments. This technology can provide better reconstruction performance and significantly outperforms the traditional algorithms in terms of IN and resolution and is expected to offer a general algorithm for brain injury monitoring imaging via EIT.

## 1. Introduction

Stroke is a major cause of clinical brain injury and is the second most common cause of death in the world. Stroke-induced brain injury mainly includes cerebral hemorrhage and cerebral ischemia [1]. Early detection and intervention are necessary to minimize the risks of death and disability caused by brain injury. Currently, clinical imaging devices such as CT and MRI can detect brain injury; however, these imaging techniques are not suitable for real-time bedside monitoring and brain injury risk warning and its complex processes [2].

Electrical impedance tomography (EIT) is a medical function imaging technique that can reconstruct impedance changes or their distribution in a domain based on simulated current and measured voltage. EIT is a low-cost, non-radiation, and real-time imaging technique. Therefore, it has been used in functional lung imaging [3,4,5,6,7,8], breast cancer detection [9,10], and brain injury monitoring [11,12,13]. Owing to variability in the electrical resistivity of tissues in the cranial brain and the changes in tissue resistivity following brain injury, EIT can enable dynamic bedside monitoring and risk, warning of brain injury [14].

Fu et al. were the first to monitor variations in impedance during dehydration treatment with intravenous infusion of mannitol in patients and observed significant changes in impedance before and after infusion [15]. Yang et al. used EIT to monitor the impedance change during mannitol infusion as part of dehydration treatment and compared the results with intracranial pressure (ICP). They reported that dehydration-induced impedance changes in EIT images and ICP change showed a close negative correlation [16,17]. Li et al. reported that brain impedance gradually increased during the total aortic arch replacement treatment and probably correlated negatively to perfusion flow [18,19]. These studies confirm the utility of EIT as an effective clinical imaging tool for brain injury monitoring.

In clinical EIT monitoring, image reconstruction algorithms have been a core topic of interest to researchers. At present, the damped least-squares (DLS) algorithm is used in clinical studies [20,21]. Liu et al. proposed an iterative DLS (IDLS) method for the simultaneous monitoring of hemorrhage and secondary ischemia. They reported that the IDLS algorithm enhanced contrast and concurrently reconstructed bidirectional disturbance targets [22]. In addition, Li et al. proposed and optimized an algorithm for large intracranial conductivity perturbations during clinical dehydration to reduce reconstruction errors and image noise (IN) over a large target area [23]. Cao et al. proposed a novel time-difference EIT algorithm using multifrequency information to enhance reconstruction quality; however, the algorithm could be used only in cases where spectral constraints were known [24]. Although the aforementioned algorithms improve the image quality of brain EIT in specific applications, they are essentially based on the DLS algorithm, which uses a parametric smoothing regularization strategy. Therefore, these methods usually cause blurred reconstructions at lower resolutions and generate more artifacts that increase the IN level and reduce the resolution of the adjacent targets.

By contrast, total variation (TV) regularization provides better boundary preservation and improves the quality of EIT images. However, it is only applicable to regularly shaped imaging regions [25]. TV is difficult to apply to the existing clinical brain EIT monitoring imaging methods because brain imaging regions are often irregular. Although other algorithms, such as structure-aware sparse Bayesian Learning (SA-SBL) [26] and sparse representation (SR) [27] can obtain sparser results, both SA-SBL and SR need to divide the sensing region into square grids for the inverse problem, which is difficult to apply in brain regions with irregular shapes. Liu et al. proposed a series of parameter-level set-based methods [28,29] and shape-driven methods [30,31] for EIT reconstruction. However, these algorithms, which primarily improve imaging performance for lung boundary shapes, have not been applied to brain EIT studies. Although numerous deep learning techniques have been proposed for EIT, the majority of these techniques are supervised and strongly rely on datasets, and there is no universal EIT dataset for monitoring brain injury [32,33,34].

Recently, with the significant success of compressed perception theory, attention has been paid to the problem of sparse regularization based on *L*_1_ parametrization, which facilitates the reconstruction of high-resolution imaging from small amounts of data [35]. Goldstein and Osher proposed the split Bregman (SB) algorithm to solve the sparse regularization problem [36]. This algorithm has been used in brain injury imaging in magnetic induction tomography [37], capacitance tomography [38], and sparse image reconstruction [39]. Wang et al. applied the SB algorithm to EIT study and demonstrated through numerical simulations that $L_{1}$ regularization provides better image quality and robustness to noise when compared with $L_{2}$ smoothing, and TV regularization [40]. However, this method requires the adjustment of two regularization parameters, making its clinical application difficult. Therefore, the development of new EIT monitoring imaging algorithms suitable for monitoring brain injuries under clinical conditions that can reduce IN and improve target resolution have been a research hotspot in this field.

In this paper, the EIT reconstruction problem is described as a least absolute shrinkage and selection operator (LASSO) model based on the sparse reconstruction method. A fast iterative shrinkage-thresholding algorithm with continuation (FISTA-C) is proposed for brain injury monitoring imaging based on EIT. Simulations using a three-dimensional (3D) human head model and physical head phantom experiments were performed to validate the performance of the proposed algorithm. The performance of the proposed method was compared with those of DLS, SB, and the fast iterative shrinkage-thresholding algorithm (FISTA). The imaging performances of all these imaging algorithms were evaluated in terms of IN and resolution.

## 2. Methods

EIT for brain injury monitoring is a real-time method to reconstruct the distribution of impedance changes Δρ using the changes Δu between the foreground measurement voltage $u_{fore}$ and background measurement voltage $u_{back}$. For a small perturbation of Δρ, the relationship between the Δu and Δρ can be linearized as

(1) Δu=SΔρ+e

where S is the sensitivity matrix, and e is the measurement noise. In this study, we used the normalized voltage changes, $\Delta u=\left(u_{fore}-u_{back}\right)./u_{back}$ where ·./· is the element-wise division operator [41].

Because the EIT inverse problem is ill-conditioned, Equation (1) cannot be directly inverted. Generally, an objective equation is constructed by introducing a regularization term, and then the optimal solution that satisfies the objective equation is obtained.

### 2.1. DLS Algorithm for Brain EIT

Based on the DLS algorithm, we can construct the objective equation as

(2) $$\Delta \tilde{\rho }=\arg\min\left(\frac{1}{2}{\left\|\Delta u-S\Delta \rho \right\|}_{2}^{2}+\frac{\lambda }{2}{\left\|R(\Delta \rho )\right\|}_{2}^{2}\right)$$

where R is the regularization matrix, λ is the regularization parameter, and $\Delta \tilde{\rho }$ is the optimal estimate of the impedance change. Solving Equation (2), we get

(3) $$\Delta \tilde{\rho }={\left(S^{T}S+\lambda R\right)}^{-1}S^{T}\Delta u$$

In this study, we used the standard Tikhonov method to construct the regularization matrix for DLS by setting $R=\mathrm{diag}(S^{T}S)$. The L-curve method was used to set the regularization parameter λ.

### 2.2. SB Algorithm for Brain EIT

The SB algorithm essentially uses the $L_{1}$ norm as the regularization term in Equation (2) and the $L_{1}$ regularized objective equation for EIT as follows:

(4) $$\Delta \tilde{\rho }=\arg\min\left({\left\|\Delta u-S\Delta \rho \right\|}_{2}^{2}+\alpha {\left\|\Delta \rho \right\|}_{1}\right)$$

where α is the regularization parameter. The framework of SB mainly includes the following steps:

(5) $$\Delta \rho^{\left(k\right)}=\arg\min(\frac{1}{2}{\left\|\Delta u-S\Delta \rho \right\|}_{2}^{2}+\frac{\beta }{2}{\left\|d^{\left(k\right)}-\Delta \rho -b_{d}^{\left(k\right)}\right\|}_{2}^{2})$$

(6) $$d^{\left(k+1\right)}=\arg\min(\alpha {\left\|d\right\|}_{1}+\frac{\beta }{2}{\left\|d-\Delta \rho^{\left(k+1\right)}-b_{d}^{\left(k\right)}\right\|}_{2}^{2})$$

(7) $$b_{d}^{\left(k+1\right)}=b_{d}^{\left(k\right)}+\Delta \rho^{\left(k+1\right)}-d^{\left(k+1\right)}$$

where β is the splitting parameter. In Equation (5), we can solve it by solving the quadratic optimization problem. Equation (6) can be easily solved by the shrinkage operator.

### 2.3. FISTA Algorithm for Brain EIT

Similar to the SB algorithm, this study also solves the EIT inverse problem using the $L_{1}$ regularized objective Equation (4). Let $f(\Delta \rho )={\left\|\Delta u-S\Delta \rho \right\|}_{2}^{2}$. Then we can transform Equation (4) into the following LASSO model:

(8) $$\arg\min\left(f(\Delta \rho )+\alpha {\left\|\Delta \rho \right\|}_{1}\right)$$

According to the definition, f(Δρ) is convex and smooth and satisfies the Lipschitz continuity condition. Then, we can obtain the iterative solution steps for the problem (8) using the gradient algorithm [42]:

(9) $$\Delta \rho^{\left(k\right)}=\arg\min(\frac{L}{2}{\left\|\Delta \rho -\left(\Delta \rho^{\left(k-1\right)}-\frac{1}{L}\nabla f(\Delta \rho^{\left(k-1\right)})\right)\right\|}_{2}^{2}+\alpha {\left\|\Delta \rho \right\|}_{1})$$

where ∇f(·) represents the gradient of f(·), k is the number of iterations, and L describes the step size. By separating the $L_{1}$ norm in Equation (9), we can solve the problem using the following iterative framework:

(10) $$q^{\left(k\right)}=S_{2\alpha /L}\left(\Delta \rho^{\left(k-1\right)}-\frac{1}{L}\nabla f(\Delta \rho^{\left(k-1\right)})\right)$$

(11) $$t^{\left(k\right)}=\frac{1+\sqrt{1+4{\left(t^{\left(k-1\right)}\right)}^{2}}}{2}$$

(12) $$\Delta \rho^{\left(k\right)}=q^{\left(k\right)}+(\frac{t^{\left(k-1\right)}-1}{t^{\left(k\right)}})\left(q^{\left(k\right)}-q^{\left(k-1\right)}\right)$$

In Equation (10), $S_{\tau }$ is the threshold operator, which is defined as

(13) $$S_{\tau }(x)=\left\{ \begin{array}{ll} x-\frac{x}{\left|x\right|}\tau & ,\left|x\right|\geq \tau \\ 0 & ,\left|x\right|<\tau \end{array}\right.$$

where τ is the threshold parameter, which is set to τ=2α/L in this study. We set L to be the Lipschitz constant of ∇f(Δρ), which is the maximum eigenvalue of $S^{T}S$.

The detailed implementation of FISTA is summarized in the following calculation process (Algorithm 1).
**Algorithm 1:** FISTA for EIT**Inputs**: A Lipschitz constant L, α, Δu, and S**Set**: k←1, $q^{\left(1\right)}=q^{\left(0\right)}=\Delta \rho^{\left(0\right)}=0$, $t^{\left(0\right)}=1$**while** convergence criteria not satisfied, **do**$q^{\left(k\right)}=S_{2\alpha /L}\left(\Delta \rho^{\left(k-1\right)}-\frac{2}{L}S^{T}(S\Delta \rho^{\left(k-1\right)}-\Delta u)\right);$update $t^{\left(k\right)}$ using Equation (11);update $\Delta \rho^{\left(k\right)}$ using Equation (12);k←k+1**end****Outputs:**$\Delta \rho^{\left(k\right)}$

To satisfy the requirements of real-time imaging in a clinical setting, the maximum number of iterations of FISTA was set to 300 in this study. The imaging results were obtained within 1 s.

### 2.4. FISTA-C Algorithm for Brain EIT

In this study, we adopted a continuation strategy to optimize the FISTA algorithm, called FISTA-C. The proposed method generates a decreasing sequence of regularization parameters that converge to zero and are used in each iteration of the FISTA calculation. First, we set an initial regularization parameter $\alpha_{1}$, $\alpha_{1}=\tilde{\alpha }{\left\|\Delta u\right\|}_{2}{\left\|S^{T}\Delta u\right\|}_{2}/L$, and update a sequence of regularization parameters $\alpha_{k+1}$ during the k-th iteration using the following equation:

(14) $$\begin{array}{l} \alpha^{\prime }=\tilde{\alpha }{\left\|\Delta u\right\|}_{2}{\left\|\Delta \rho^{\left(k\right)}-\Delta \rho^{\left(k-1\right)}\right\|}_{2}\\ \alpha_{k+1}=\left\{ \begin{array}{l} \alpha^{\prime },\;\alpha^{\prime }<\gamma \alpha_{k}\\ \alpha_{k},\;\alpha^{\prime }\geq \gamma \alpha_{k} \end{array}\right. \end{array}$$

where $\tilde{\alpha }>0$ and γ∈(0,1) are constants, $\Delta \rho^{\left(k\right)}$ and $\Delta \rho^{\left(k-1\right)}$ are the impedance changes during the k-th and k−1-th iterations, respectively, and Δu represents the change in the boundary measurement voltage vector. A detailed implementation of the proposed FISTA-C for EIT is summarized in the following computational procedure (Algorithm 2).
**Algorithm 2:** FISTA-C for EIT**Inputs**: A Lipschitz constant L, $\tilde{\alpha }>0$, γ>0, Δu, and S**Set**: k←1, $q^{\left(1\right)}=q^{\left(0\right)}=\Delta \rho^{\left(0\right)}=0$, $t^{\left(0\right)}=1$, and $\alpha_{1}=\tilde{\alpha }{\left\|\Delta u\right\|}_{2}{\left\|S^{T}\Delta u\right\|}_{2}/L$**while** convergence criteria not satisfied, **do**$q^{\left(k\right)}=S_{\alpha_{k}/L}\left(\Delta \rho^{\left(k-1\right)}-\frac{1}{L}\nabla f(\Delta \rho^{\left(k-1\right)})\right);$update $t^{\left(k\right)}$ using Equation (11);update $\Delta \rho^{\left(k\right)}$ using Equation (12);update $\alpha_{k+1}$ using Equation (14);k←k+1**end****Outputs:**$\Delta \rho^{\left(k\right)}$

When compared with FISTA, although FISTA-C has an additional update rule of the regularization parameter given in Equation (14), it has little impact on the execution speed of the algorithm. On the contrary, a previous study has reported that the convergence of the FISTA algorithm can be accelerated by this strategy [43]. Although the selection of parameters for the algorithm is difficult in practical applications, in this study, we set the initial parameters as $\tilde{\alpha }=0.5/(100\cdot \max(S^{T}\Delta u))$ and γ=0.9 for FISTA-C in all experiments. Consistent with FISTA, the maximum number of iterations for FISTA-C was also set to 300 to ensure that we obtained imaging results within 1 s and met the requirements for real-time brain monitoring.

### 2.5. Experimental Framework

#### 2.5.1. Evaluation Metrics for Image Quality

During brain EIT image monitoring, artifacts in the imaging frames can significantly affect the diagnosis of the disease. Therefore, minimizing the artifacts is crucial. Referring to the objective quantifiers proposed by Adler et al. [44], we used IN and resolution (RES) to evaluate the performance of the algorithm. In this study, we defined the region of interest (ROI) as the region that exceeded 50% of the maximum change in reconstruction impedance in reconstruction results [45].

IN indicates the degree of artifacts or noise outside the ROI. It is defined as the contrast noise ratio between the ROI and background:

(15) $$\mathrm{IN}=\frac{\sqrt{\frac{1}{N_{B}-1}\sum_{i\in B}\left(\Delta \rho_{i}-\mathrm{mean}\left(\Delta \rho^{B}\right)\right)}}{\left|\mathrm{mean}\left(\Delta \rho^{ROI}\right)-\mathrm{mean}\left(\Delta \rho^{B}\right)\right|}$$

where mean(·) denotes the mean value, B represents the background region (i.e., the region outside the ROI), $N_{B}$ is the number of elements in B, and $\Delta \rho^{ROI}$ and $\Delta \rho^{B}$ denote the reconstruction impedance variations in the ROI and background, respectively.

RES is a metric equivalent to the size of the point spread function and is the ratio of the estimated area of the ROI to the area of the entire imaging area, also known as the blur radius [44,46].

(16) $$\mathrm{RES}=\sqrt{A_{\mathrm{ROI}}/A_{0}}$$

where $A_{\mathrm{ROI}}$ denotes the area of the ROI and $A_{0}$ denotes the area of the entire imaging region. To more accurately represent the shape of the target distribution, RES must be small and uniform. A smaller RES represents the ability to distinguish between closer targets [44]. Considering that the uniformity of RES is more important than its value for the evaluation of EIT images, the standard deviation of RES was calculated in this study to evaluate the uniformity of RES.

#### 2.5.2. Numerical Simulations

**Figure 1:**
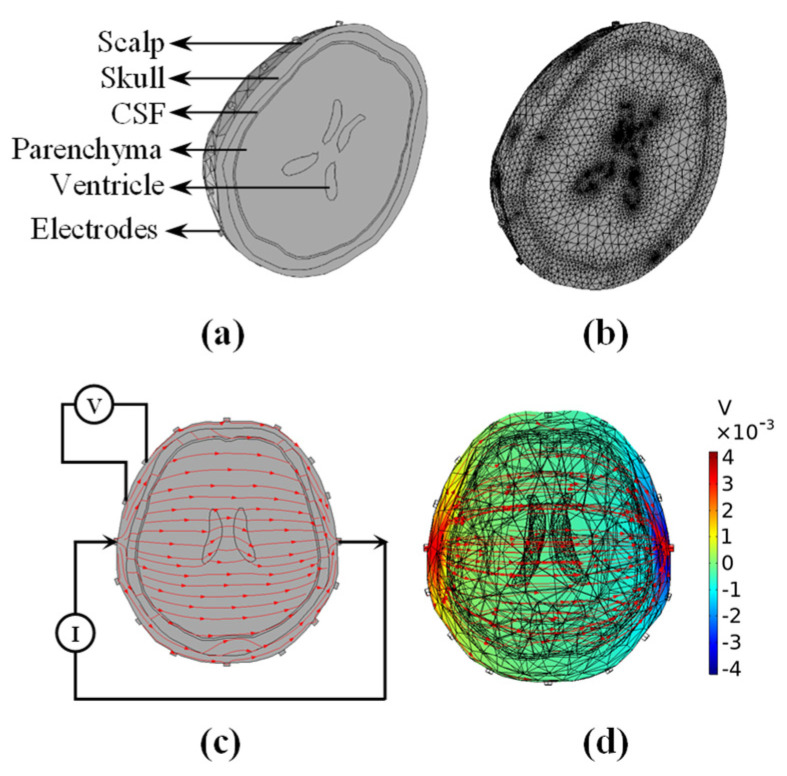
Brain EIT model used for simulation experiment. (**a**) 3D realistic head. (**b**) Discrete finite element mesh. (**c**) A polar drive pattern and red flow lines with arrows represent the current direction. (**d**) Intracranial potential distribution (pseudo-color) and current density lines (red flow lines) under single excitation. (CSF: cerebrospinal fluid).

To evaluate and compare the performances of various algorithms during EIT brain hemorrhage monitoring, we designed a realistic 3D brain model in COMSOL. The model contained the scalp, skull, cerebrospinal fluid, brain parenchyma, and ventricles. Sixteen electrodes were placed uniformly around the head to form the brain EIT simulation model depicted in Figure 1a. Next, we set the conductivity of the skull referring to the resistivity of the standard three-layer skull reported by Tang et al. [47] and the conductivity of other tissue in the model referring to Gilad et al. [48] (see Table 1). After constructing the simulation model, finite element discretization was performed to obtain a finite element model containing approximately 173,100 tetrahedral elements, as depicted in Figure 1b. In this study, we used polar drive patterns, which are widely used in brain EIT (see Figure 1c) [49,50] and set the injected normal current density in the simulation model to 1 A/m^2^. Figure 1d illustrates the intracranial potential distribution and current density flow lines under a single excitation.

**Figure 2:**
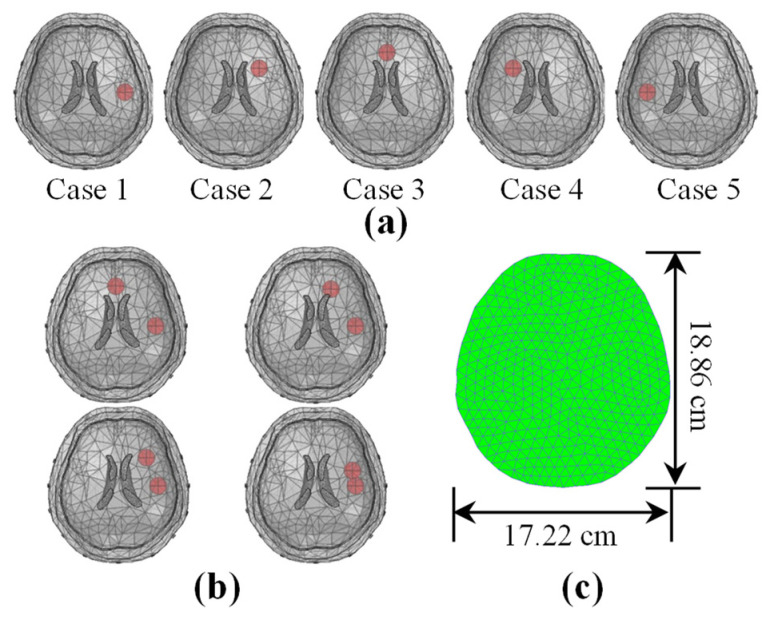
The model used in simulation experiments. (**a**) 5 mL spherical target at various locations in a 3D head model. (**b**) Two 5 mL spheres at distances of 7.07, 5.56, 3.83, and 1.95 cm, respectively. (**c**) Finite element model for the inverse problem.

In the simulation experiments, the hemorrhagic lesions were simulated by adding 5 mL spheres to the 3D brain model with conductivity (see Table 1). First, we obtained the voltage data without lesions as the background data $u_{back}$. Next, we measured the voltage data with lesions as the foreground data $u_{fore}$ and computed the normalized voltage changes Δu for image reconstruction. We added a 5 mL spherical target simulated lesion at various locations in the 3D head model (see Figure 2a) to evaluate the performance of the algorithm at various locations. Next, we examined the imaging performance of the algorithm at various noise levels. Foreground measurements $u_{fore}$ were obtained by setting a 5 mL sphere as a lesion at 5 cm anterior in the 3D head. Noise with the same signal-to-noise ratio (SNR) was added to both $u_{back}$ and data $u_{fore}$. We defined the SNR as follows:

(17) $$\mathrm{SNR}=20\log_{10}\left({\left\|u\right\|}_{2}^{2}/{\left\|n\right\|}_{2}^{2}\right)\;$$

where u and n are the measured voltage and noise vectors, respectively. Finally, to further evaluate the imaging resolution of the algorithm, two 5 mL spheres at distances of 7.07, 5.56, 3.83, and 1.95 cm were set in the simulation model to simulate double hemorrhages (see Figure 2b). For EIT image reconstruction, a 2D finite element model, which contained 771 triangular elements and 416 nodes, matching the plane of the electrode, was used (see Figure 2c).

#### 2.5.3. Head Phantom Experiments

**Figure 3:**
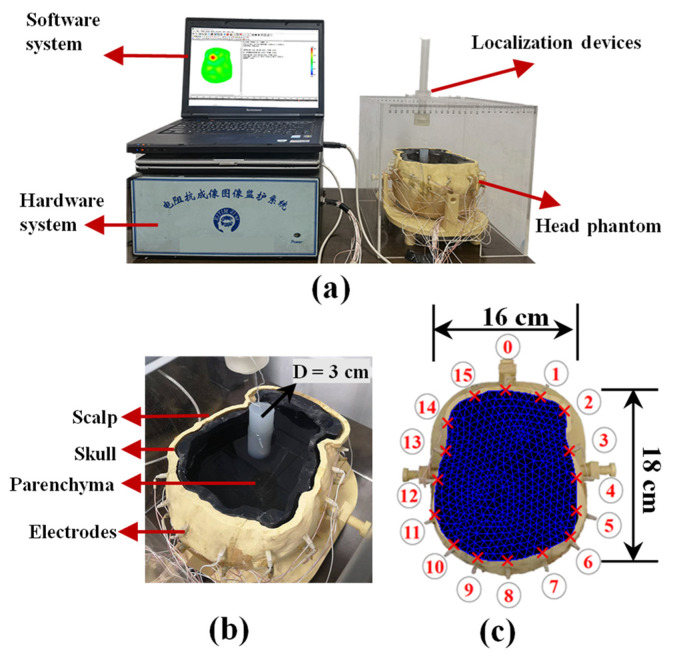
Head phantom experimental setup. (**a**) Head phantom experimental platform. (**b**) Head phantom model with actual anatomical structure and conductivity distribution containing 16 electrodes and cylindrical agar block as a simulated lesion. (**c**) Finite element model for reconstruction, matching the plane of the electrode in the head phantom. (D: diameter; “red crosses”: electrode locations; “circled numbers”: electrode numbers).

To further verify the imaging performance of the algorithm in practical applications, a head phantom model experiment was established in this study. The phantom experimental data were obtained through the FMMU-EIT5 data acquisition system [51]. Figure 3a depicts the head phantom experimental platform, in which the software system runs on a computer with a 2.16 GHz CPU and 4 GB RAM. In this experiment, we adopted a polar pattern to drive the hardware system and set the amplitude of the excitation current to 0.25 mA at 50 kHz. Figure 3b depicts the head phantom model used for the experiment, which was fabricated by a 3D printer, has actual anatomical structure and conductivity distribution [52], and contains 16 electrodes. Consistent with Figure 2a,b, in the computer simulation experimental setup, single- and dual-target disturbance models were established at various positions. After obtaining the voltage variation data, we used the finite element model depicted in Figure 3c, matching the electrode plane in the head phantom model, to reconstruct the conductivity change, which contained 832 triangular elements and 450 nodes.

## 3. Results

### 3.1. Numerical Simulations

**Figure 4:**
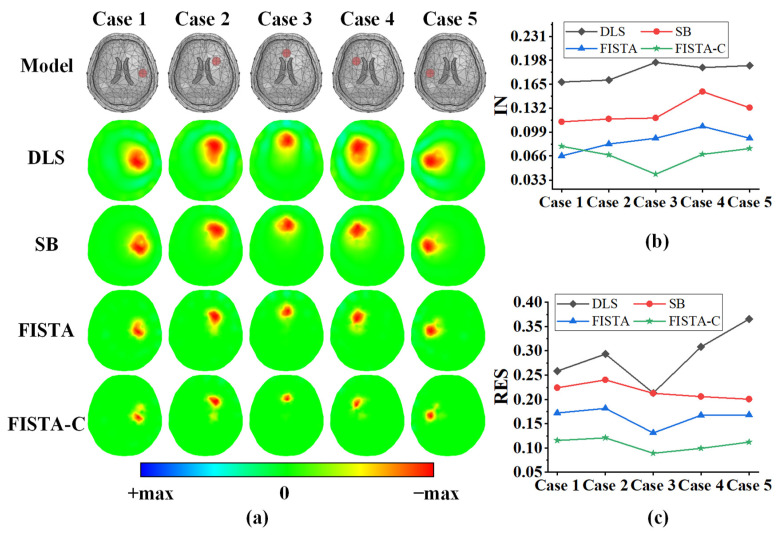
Reconstruction results of a single lesion target at various locations in the 3D head model under noise-free conditions. (**a**) Simulation model and imaging results of DLS, SB, FISTA, and FISTA-C. (**b**) Image noise (IN) analysis for imaging results of the four algorithms. (**c**) Resolution of imaging results of the four algorithms.

Figure 4a depicts the imaging results for a single target at various positions in the 3D head model. The first row in this figure is the simulation model setup, and the second to fifth rows are reconstruction results of DLS, SB, FISTA, and FISTA-C, respectively. We did not add noise to the data used for imaging. The parameters of the DLS algorithm were determined using the L-curve method in all experiments. The splitting parameter in the SB algorithm was set to 0.01 and the regularization parameter was set to 0.001. The regularization parameter of the FISTA algorithm was set to $\lambda =1\times 10^{-5}$. The parameters of the FITSA-C algorithm did not require additional settings, as discussed in Section 2.3. The results in Figure 4a indicate that all four algorithms can reflect the real position of the lesion. The DLS algorithm reconstructs a blurred target edge and ring artifacts. The SB algorithm improves it to some extent but is not as effective as FISTA and FISTA-C. We calculated the evaluation metrics of the reconstructed images. The IN and resolution results are depicted in Figure 4b,c, respectively. Table 2 shows the mean of IN and the mean and standard deviation of RES for the imaging results of the four algorithms.

As shown in Figure 4b, FISTA-C and FISTA have lower image reconstruction noise than DLS and SB. As can be seen from Table 2, FISTA-C can reduce IN by 63.2%, 47.2%, and 29.9% compared with the DLS, SB, and FISTA algorithms, respectively. Figure 4c depicts that FISTA-C and FISTA have smaller RES values than those of the DLS and SB algorithms. The smaller RES represents how close two targets can be distinguished. In addition, it can be seen from Table 2 that FISTA-C not only has the smallest RES among the four algorithms but also has the lowest standard deviation. This indicates that FISTA-C has the best uniformity of RES among the four algorithms.

**Figure 5:**
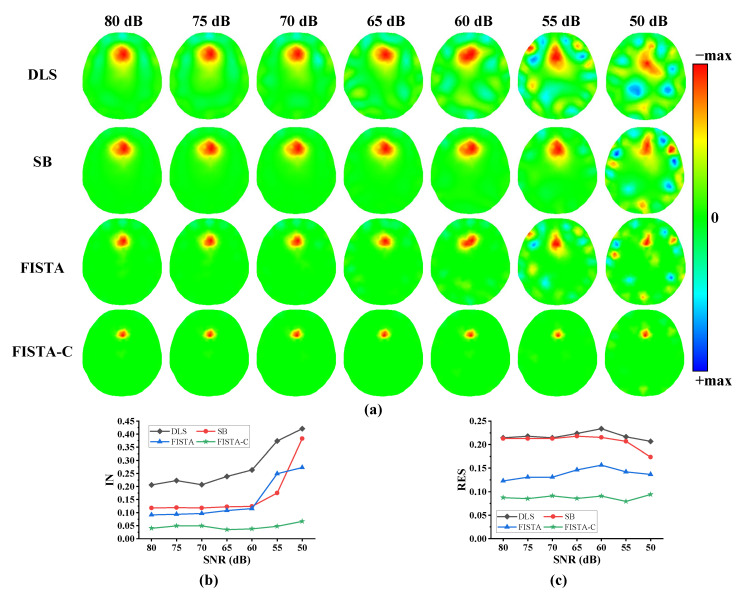
Analyses of reconstruction performances of four algorithms under various noise levels. (**a**) The first to fourth rows are imaging results of DLS, SB, FISTA, and FISTA-C, respectively, with an SNR of 80–50 dB. (**b**) Image noise (IN) analyses of imaging results of the four algorithms. (**c**) Resolutions of imaging results for the four algorithms. (std: standard deviation).

To further verify the imaging performance of the proposed algorithm under various noise levels, a 5 mL spherical target was set at 5 cm anterior in the 3D model of the human head to simulate the lesion. Next, Gaussian white noise with an SNR of 80–50 dB was added to the measurement data. The final imaging results are depicted in Figure 5. We set the splitting and regularization parameters in the SB algorithm to 0.005, and $\lambda =5\times 10^{-5}$ in FISTA when the SNR was 50 dB. In the remaining cases, the algorithm parameter settings were the same as in the first experiment. Figure 5a shows that DLS, SB, and FISTA have significantly more image noise when the noise level gradually increases, whereas FISTA-C exhibited less image noise. The analyses of IN and resolution of the imaging results are presented in Figure 5b,c, respectively. Figure 5b indicates that FISTA-C and FISTA algorithms have lower image reconstruction noise than the DLS and SB algorithms. As can be seen from Table 3, FISTA-C can reduce IN by 83.3%, 72.3%, and 68.7% compared with the DLS, SB, and FISTA algorithms, respectively. Figure 5c and Table 3 depict that FISTA-C has the smallest RES and standard deviation values when compared with those of the DLS, SB iteration, and FISTA algorithms, indicating the best uniformity.

**Figure 6:**
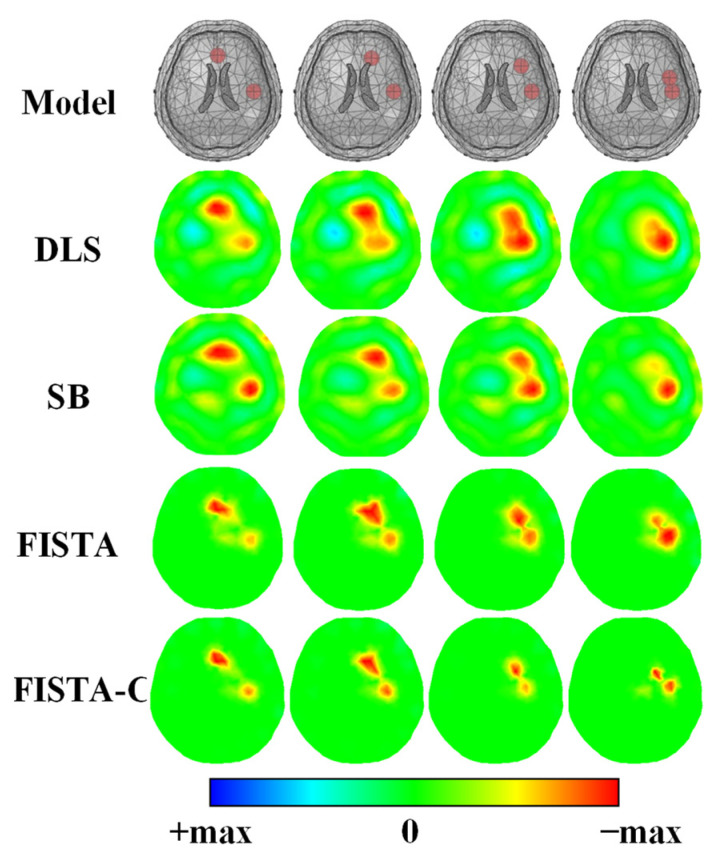
Reconstruction results of a double-lesion target with distances of 7.07, 5.56, 3.83, and 1.95 cm.

The results of the simulation experiments suggest that FISTA-C has the best resolution. Therefore, the simulation experiment for dual targets was set up referring to Figure 2b to verify further the ability of FISTA-C to distinguish near targets. Figure 6 shows the imaging results of the two targets getting closer together. We set the splitting and regularization parameters in the SB algorithm to $\beta =2\times 10^{-6}$ and $\alpha =1\times 10^{-6}$, respectively, referring to [40] and $\lambda =1\times 10^{-5}$ in FISTA. We added white Gaussian noise with an SNR of 60 dB to the measurement data.

As is evident from Figure 6, all four algorithms can identify both targets when they are far away. However, when the distance between the two reduces, DLS, SB, and FISTA cannot identify the targets, whereas FISTA-C can still distinguish the targets in the imaging results. The simulation results suggest that FISTA-C has a better ability to distinguish the closer targets and can reconstruct double targets even when two 5 mL spheres are 1.95 cm apart.

### 3.2. Head Phantom Experiments

**Figure 7:**
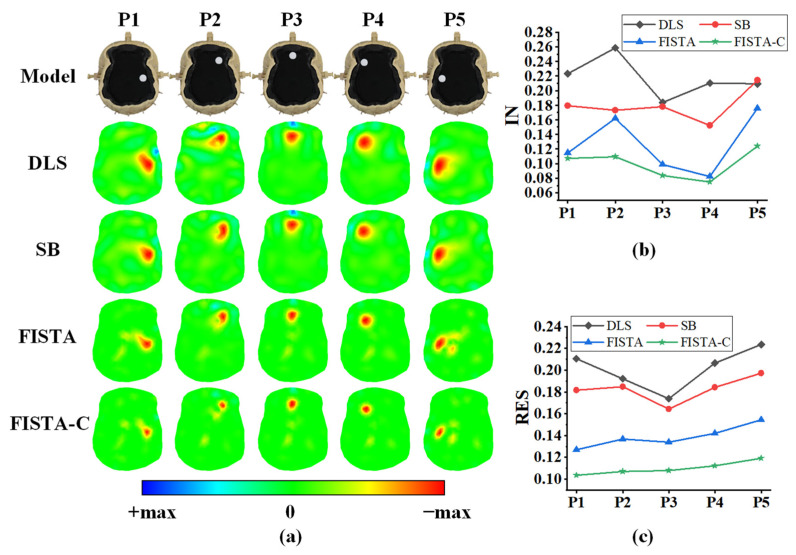
Reconstruction results of a single target at various locations in the head phantom model. (**a**) Phantom model and imaging results of DLS, SB, FISTA, and FISTA-C, respectively. (**b**) Image noise (IN) analysis of imaging results of the four algorithms. (**c**) Resolutions of imaging results for the four algorithms.

The imaging results of the single-target phantom experiments are depicted in Figure 7a, where the first row shows the setup of the phantom model, and the second to fifth rows the DLS, SB, FISTA, and FISTA-C imaging results, respectively. In this experiment, we set the splitting and regularization parameters in the SB algorithm to 0.0001 and 0.001, respectively, and set $\lambda =1\times 10^{-4}$ in FISTA. The results in Figure 7a suggest that all four algorithms can reconstruct the location of a single target accurately.

The quantitative IN analysis of the imaging results are presented in Figure 7b and Table 4, which indicates that FISTA-C has the lowest image reconstruction noise, and FISTA-C reduces IN by 54.4%, 44.7%, and 22.7% when compared with those of DLS, SB, and FISTA, respectively. The resolution analysis of the imaging results in Figure 7c and Table 4 suggest that FISTA-C has the smallest RES and standard deviation among the four algorithms, indicating that this algorithm has the best uniformity of RES among the four algorithms.

**Figure 8:**
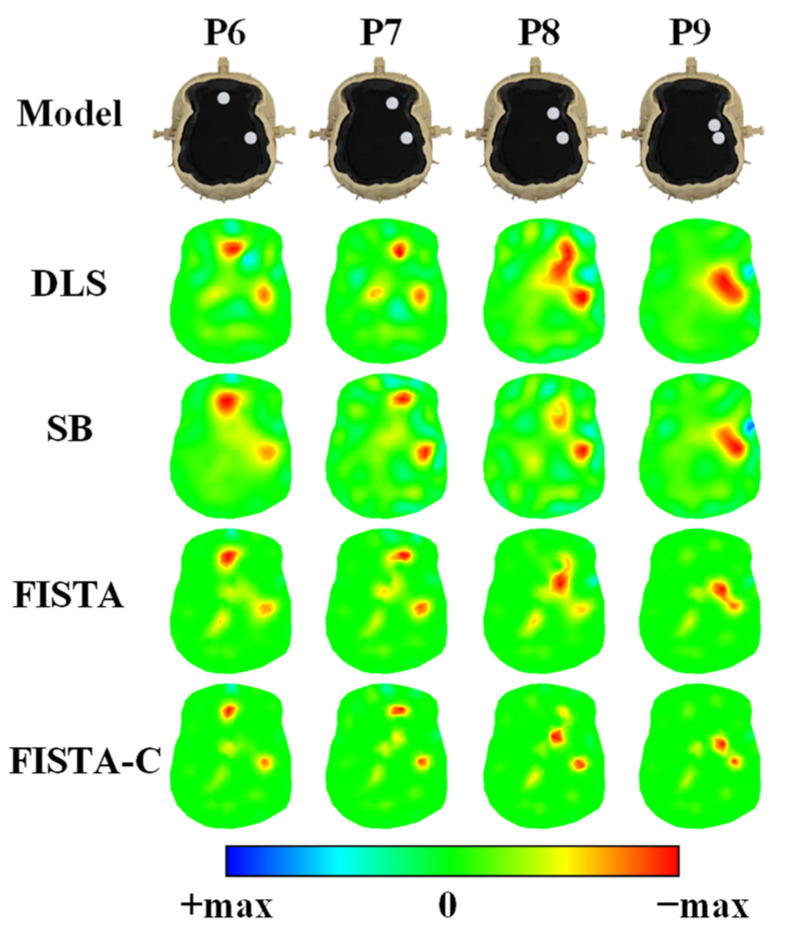
Reconstruction results of a dual-target with different distances in head phantom experiments.

Consistent with the simulation experiment, a two-target experiment was set up for the physical model experiment to verify further the ability of FISTA-C to distinguish closer targets. In this experiment, we set the splitting and regularization parameters in the SB algorithm to 0.001 and 0.05, respectively, and set $\lambda =1\times 10^{-4}$ in FISTA. As is evident from Figure 8, all four algorithms can identify both targets when the targets are separated from one another. However, when the distance between the targets is reduced, the DLS and SB algorithms cannot identify the targets, whereas the FISTA and FISTA-C algorithms can still distinguish the targets in the imaging results. However, the results of FISTA were not as distinct as those of FISTA-C. The head phantom experiment results suggest that FISTA-C has a better ability to distinguish closer targets and can reconstruct double targets even when the two targets are close together.

## 4. Discussion

To reduce IN and improve the resolution of closer targets in brain injury monitoring imaging using EIT, this study modeled the brain EIT problem as a LASSO model based on a sparse reconstruction method and proposed an optimization algorithm based on a continuous strategy, FISTA-C. Numerical simulation experiments were performed by constructing a 3D cranial model to verify that FISTA-C achieved the lowest IN and the best spatial resolution when compared with DLS, SB, and FISTA. Physical head model experiments also confirmed that FISTA-C performs better and is more suitable for EIT-based brain injury monitoring imaging.

On the one hand, FISTA-C is based on the $L_{1}$ norm regularization method; therefore, the imaging results are sparser than those of the $L_{2}$ regularization method. This will make the area outside the target as close to zero as possible, which can effectively reduce IN and improve spatial resolution. On the other hand, compared with FISTA, SB, and other algorithms, the FISTA-C can obtain better imaging without excessive parameter adjustment. Unlike the common use of a fixed descent rate to generate a decreasing sequence, FISTA-C utilizes the $\left\{\Delta \rho^{\left(k\right)}\right\}$ sequence generated by FISTA to generate a decreasing sequence $\left\{\alpha_{k}\right\}$ of regularization parameters. The subsequence $\left\{\alpha_{k}\right\}$ converges to zero, which forces the equality constraint and eventually leads FISTA-C to solve the basis pursuit problem [43]. This enables FISTA-C to obtain high-quality reconstruction results without excessive parameter adjustment. It makes the proposed algorithm more favorable for application in clinical studies. Recently, some studies have proposed algorithms based on iterative optimization for the difficulty in parameter selection in practical studies [53]. Others have proposed the fidelity-embedded regularization method [54]. In the future, we plan to consider combining these approaches to obtain better reconstruction performance.

Although the IN and RES of the imaging results were improved to some extent, the accuracy of the boundary shape of the reconstructed target still needs to be improved. This may be attributed to the reconstruction of the inverse problem using a finite element mesh of a triangular mesh. In the future, we will also investigate whether the accuracy of shape reconstruction can be further improved by combining some other algorithms, such as shape-driven algorithms [30,31].

Considering that the main contribution of this paper is the proposal and first application of FISTA-C to EIT monitoring imaging of brain injury, we emphasize the improvement in EIT image quality. The convergence analysis related to the algorithm has been analyzed and proved in detail in the references [43]; therefore, the convergence of the algorithm is not discussed in detail in the main text of this paper. Since the acquisition rate of our hardware was set at 1 fps, we set the maximum number of iterations to 300 to meet the real-time imaging requirements of brain injury monitoring. In fact, for the hardware used in this study, the runtime of FISTA-C was less than 0.6 s, which is similar to that of FISTA. From the perspective of computational complexity, compared with FISTA, FISTA-C does not increase computation significantly; the computational complexity of FISTA-C is $O(1/k^{2})$, which is the same as that of FISTA.

From the reconstruction results of the head phantom in Figure 7 and Figure 8, more artifacts are present than in the simulation experiment. This also shows the necessity of using a more accurate model for algorithm study. Although this study used a head model with the actual anatomical structure to verify the effectiveness of the proposed method, the human head has a more complex and inhomogeneous conductivity distribution [55]. In addition, compared with phantom experiments, the real signals coming from a clinical human test are usually affected by disturbances such as electrode-scalp contact impedance, patient body motion, medical staff handling, and environmental noise. This poses a greater challenge for algorithm imaging. However, there are no publicly available human databases to conduct algorithmic studies. In the future, clinical experimental studies will be the key research direction for EIT studies.

## 5. Conclusions

IN and RES are significant challenges in brain injury monitoring imaging via EIT. To address these challenges, this study modeled brain EIT imaging as a LASSO problem and proposed FISTA-C based on a continuous optimization strategy for the first time. Results of numerical simulation experiments with 3D realistic cranial models and head phantom experiments suggest that FISTA-C can provide better reconstruction performance. FISTA-C significantly outperforms DLS, SB, and FISTA in terms of IN and RES. Therefore, FISTA-C has better performance and has potential application in EIT imaging for monitoring brain injury. However, the accuracy of reconstructing the boundary shape needs to be improved, which can be done in the future by combining it with a shape-driven algorithm.

In addition, FISTA-C is more practical in clinical application studies because it requires fewer adjustments to algorithm parameters. In the future, the validation of the algorithm in animal experiments and clinical trials is necessary to explore its efficacy in addressing the challenges in existing EIT brain monitoring imaging methods.

## Figures and Tables

**Table 1:** Conductivity setting of different tissue.

Tissue	Conductivity σ (S/m)
Scalp	0.44
Skull	0.012
Parenchyma	0.149
Cerebrospinal fluid	1.79
Ventricle	1.79
Lesion	0.67

**Table 2:** Image noise and resolution analysis for imaging results of the four algorithms for a single lesion target at various locations in the 3D head model under noise-free conditions.

Method	IN (Mean)	RES (Mean ± Std)
DLS	0.182	0.288 ± 0.051
SB	0.127	0.217 ± 0.014
FISTA	0.087	0.164 ± 0.017
FISTA-C	0.067	0.107 ± 0.011

Note: IN, image noise; RES, resolution; std, standard deviation; DLS, damped least-squares; SB, split Bregman; FISTA, fast iterative shrinkage-thresholding algorithm; FISTA-C, FISTA with continuation.

**Table 3:** Image noise and resolution analysis for imaging results of the four algorithms under various noise levels.

Method	IN (Mean)	RES (Mean ± Std)
DLS	0.276	0.218 ± 0.008
SB	0.166	0.207 ± 0.014
FISTA	0.147	0.139 ± 0.010
FISTA-C	0.046	0.088 ± 0.004

Note: IN, image noise; RES, resolution; std, standard deviation; DLS, damped least-squares; SB, split Bregman; FISTA, fast iterative shrinkage-thresholding algorithm; FISTA-C, FISTA with continuation.

**Table 4:** Image noise and resolution analysis for imaging results of the four algorithms for a single target at various locations in the head phantom model.

Method	IN (Mean)	RES (Mean ± Std)
DLS	0.217	0.201 ± 0.017
SB	0.179	0.182 ± 0.011
FISTA	0.128	0.139 ± 0.009
FISTA-C	0.099	0.109 ± 0.005

Note: IN, image noise; RES, resolution; std, standard deviation; DLS, damped least-squares; SB, split Bregman; FISTA, fast iterative shrinkage-thresholding algorithm; FISTA-C, FISTA with continuation.

## Data Availability

Not applicable.
